# Interaction Effect of Social Isolation and High Dose Corticosteroid on Neurogenesis and Emotional Behavior

**DOI:** 10.3389/fnbeh.2017.00018

**Published:** 2017-02-21

**Authors:** Jackie N.-M. Chan, Jada C.-D. Lee, Sylvia S. P. Lee, Katy K. Y. Hui, Alan H. L. Chan, Timothy K.-H. Fung, Dalinda I. Sánchez-Vidaña, Benson W.-M. Lau, Shirley P.-C. Ngai

**Affiliations:** ^1^Department of Rehabilitation Sciences, The Hong Kong Polytechnic UniversityHong Kong, Hong Kong; ^2^Department of Ophthalmology, The University of Hong KongHong Kong, Hong Kong

**Keywords:** neurogenesis, hippocampus, social isolation, corticosterone, emotion, mood

## Abstract

Hypercortisolemia is one of the clinical features found in depressed patients. This clinical feature has been mimicked in animal studies via application of exogenous corticosterone (CORT). Previous studies suggested that CORT can induce behavioral disturbance in anxious-depressive like behavior, which is associated with suppressed neurogenesis. Hippocampal neurogenesis plays an important role in adult cognitive and behavioral regulation. Its suppression may thus lead to neuropsychiatric disorders. Similar to the effects of CORT on the animals’ depression-like behaviors and neurogenesis, social deprivation has been regarded as one factor that predicts poor prognosis in depression. Furthermore, social isolation is regarded as a stressor to social animals including experimental rodents. Hence, this study aims to examine if social isolation would induce further emotional or anxiety-like behavior disturbance and suppress neurogenesis in an experimental model that was repeatedly treated with CORT. Sprague-Dawley rats were used in this study to determine the effects of different housing conditions, either social isolated or group housing, in vehicle-treated control and CORT-treated animals. Forced swimming test (FST), open field test (OFT) and social interaction test (SIT) were used to assess depression-like, anxiety-like and social behaviors respectively. Immunohistochemistry was performed to quantify the number of proliferative cells and immature neurons in the hippocampus, while dendritic maturation of immature neurons was analyzed by Sholl analysis. Social isolation reduced latency to immobility in FST. Furthermore, social isolation could significantly reduce the ratio of doublecortin and bromodeoxyuridine (BrdU) positive cells of the neurogenesis assay under CORT-treated condition. The current findings suggested that the behavioral and neurological effect of social isolation is dependent on the condition of hypercortisolemia. Furthermore, social isolation may possibly augment the signs and symptoms of depressed patients with potential alteration in neurogenesis.

## Introduction

Major depressive disorder is well characterized to be associated with the altered feedback control of hippocampus on the hypothalamic-pituitary-adrenal (HPA) axis with an over production of glucocorticoids (Smith and Vale, 2006). Hypercortisolemia is thus a common clinical feature that has been observed in patients with depression (Axelson et al., 1993). The abnormal increase in glucocorticoid level may in turn cause further hippocampal damage thereby impairing hippocampal-related functions, such as learning and memory as observed in patients (O’Connor et al., 2000). In accord with the depressive symptoms presented by patients with depression, similar symptoms were also reported in patients receiving glucocorticoid therapy (Brown and Suppes, 1998) indicating the linkage between high levels of cortisol and depressive symptoms. Based on the clinical findings of hypercortisolemia, chronic injections of corticosterone (CORT) have been one of the adopted methods for the study of depression in rodents. It aims to mimic the hypercortisolemia featured in patients with depression. Accumulating evidence has shown that exogenous injections of CORT may produce anxiety and emotional changes in animals (Skórzewska et al., 2006). Though the face and construct validity of using this animal model may not be as high as applying the environment-induced stress model, exogenous CORT injections have been shown to produce less variable behavioral results possibly due to the standardized level of circulating CORT (Sarah et al., 2006).

Social isolation is one of the environmental factors that exacerbate depression (Sharp and Lipsky, 2002). This was supported by a long-term follow-up study in which patients with social isolation demonstrated a high risk of recurrent depressive episodes (Riise and Lund, 2001), while social interaction was reported to facilitate recovery from depression (Cheung et al., 2016). For rodents, being a social species, social isolation is regarded as a potent stressor for inducing physiological changes which include alterations in learning, aggression and reduced body weight (Beery and Kaufer, 2015). Furthermore, it has been linked to other depression related physiological outcomes such as reduced dopamine signaling (Heidbreder et al., 2000) and altered neurogenesis (Famitafreshi et al., 2015).

Adult neurogenesis primarily occurs in the subventricular zone of the lateral ventricles and the subgranular zone within dentate gyrus (DG) of the hippocampus (Ruan et al., 2014). It plays an important role in regulating cognitive and behavioral functions of adult mammals by increasing or replacing neurons in the central nervous system (Imayoshi et al., 2009). Moreover, it is regarded as one of the hypothesis for the mechanism underlying depression. Santarelli et al. (2003) have reported the requirement of adult hippocampal neurogenesis for the efficacy of antidepressants. In detailed, the study has demonstrated that with the disruption of antidepressant-induced neurogenesis, the behavioral responses to antidepressants were blocked. This suggested that adult neurogenesis is possibly related to the pathophysiology of depression and explained the reason of delayed improvement for mood-related symptoms observed in patients with antidepressant treatments (Drew and Hen, 2007; Ruan et al., 2014).

In contrast to the “neurogenesis hypothesis of depression”, several studies have shown that a direct inhibition of adult neurogenesis did not induce any depression symptoms in animals (Revest et al., 2009; Surget et al., 2011). These studies suggested that the neurobiology of depression may possibly rely on different aspects of brain functioning in which the process of adult neurogenesis should not be the only factor that contribute to the depressive symptoms (Castro et al., 2010; Lavergne and Jay, 2010). Adult neurogenesis has been suggested it can buffer stress response and depressive behaviors (Snyder et al., 2011). Hence, reduced neurogenesis may result in increased depression-like behavior when the individual is under stress (Petrik et al., 2012). Therefore, it was of our interest to investigate how different stressors are interacted in relations with adult neurogenesis in rodents with the presentations of depression and anxiety symptoms.

Injection of exogenous CORT has been well documented to reduce neurogenesis on both neurogenic sites (Lau et al., 2007; Brummelte and Galea, 2010). Furthermore, the decreased neurogenesis by CORT treatment was found to be correlated to the depressive phenotypes as reflected by increased immobility time of forced swimming test (FST) in animals (Fenton et al., 2015). Social isolation, another stressor, has previously been shown to alter the process of neurogenesis and the behavior among isolated animals (Fone and Porkess, 2008). Consistent with the reduced hippocampal neurogenesis, higher levels of anxiety was found in isolated, non-human primates (Cinini et al., 2014). In contrast to the effects of reducing neurogenesis, several studies reported that social isolation could enhance cell survival in the hippocampus. The possible explanations may be due to the different strains of animals adopted in the experiments. In addition, the enhanced cell survival was proposed to maintain neuroplasticity under stressed conditions (Taylor, 2006; Lieberwirth and Wang, 2012; Ruscio et al., 2015). However, the possible effects of social isolation on neurogenesis, particularly when under a depressed state, deserve further investigation.

The present study aims to examine if the effects of social isolation on neurogenesis and the development of both anxiety and emotional related behaviors can possibly interact with a high CORT level in rodents. It was hypothesized that social isolation would impair hippocampal neurogenesis and exacerbate depression-like and anxiety-like behaviors in animals with or without CORT treatment.

## Materials and Methods

### Animals and Grouping

The experimental protocol was approved by the Animal Subjects Ethics Sub-committee of The Hong Kong Polytechnic University. Twenty-six adult male Sprague–Dawley rats weighted 400 g ± 100 g were used in this study. The animals were kept under a 12:12-h light/dark cycle at 22°C and provided with free access to food and water. All animals were kept in laboratory cages of 2–4 animals per cage in good health condition before the experiments. Before allocating into groups, the animals were weight matched in order to minimize the group differences in body weight. Their body weight was reassessed on the last day of experiment to evaluate the change, if any, in 14 days. A decreased body weight would indicate the efficacy of CORT injection (Sarah et al., 2006; Marks et al., 2009).

**Figure 1:**
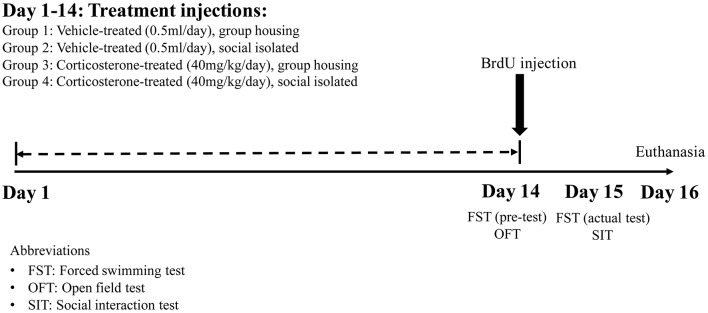
**Fourteen days treatment schedule and behavioral tests**. The group housing or social isolated conditions together with either corticosterone (CORT) or vehicle injections were carried out continuously for 14 days. The open field test (OFT) and the pre-test of forced swimming test (FST) was carried out on Day 14. The social interaction test (SIT) and the actual test of FST was performed on Day 15 followed by euthanasia the day after, Day 16. Bromodeoxyuridine (BrdU) was injected for proliferating cell tracing on Day 14.

The animals were divided into four groups, with each group receiving different treatment injections in either group housing or social isolation conditions. The four groups were: Group 1: Vehicle-treated group in group housing condition (Gp_Vehicle; *n* = 6); Group 2: Vehicle-treated group in social isolation condition (Iso_Vehicle; *n* = 6); Group 3: CORT-treated group in group housing condition (Gp_Cort; *n* = 7) and Group 4: CORT-treated group in social isolation condition (Iso_Cort; *n* = 7). Figure 1 summarizes the treatment schedule of this study.

### Treatment Injections

The chemicals for treatment injections were supplied by Sigma-Aldrich Co. (St Louis, MO, USA). Group 1 and 2 received vehicle injection (Propylene glycol; 0.5 ml/day; by subcutaneous injection), while the CORT-treated groups (Groups 3 and 4) received repeated high-dose CORT injection (40 mg/kg/day; by subcutaneous injection) that was sufficient to induce moderate to severe level of depression-like behavior in rodents as shown in previous studies (Sarah et al., 2006; Marks et al., 2009). The CORT was dissolved in propylene glycol before administration. At the last day of treatment, two dosages of bromodeoxyuridine (BrdU; 100 mg/kg) with an interval of 10 h were administered through intraperitoneal injection to label dividing cells for cell proliferation assay.

### Housing Conditions

The animals in social isolation condition (Group 2 and 4) were housed individually in laboratory cage (43 cm (L) × 27 cm (W) × 16 cm (H)) for 14 days (Abramets et al., 2005). The animals in group-housing condition (Group 1 and 3) were kept in laboratory cage of same dimension for the same period in groups of 3–4 animals per cage.

### Behavioral Tests

All of the behavioral tests were videotaped for behavioral analysis by an experimenter blinded to the animal groupings. During behavioral tests, the equipment was thoroughly cleaned between tests using a dilute 0.4% (v/v) acetic acid solution for minimizing potential olfactory cues.

#### Open Field Test (OFT)

Each animal was placed individually in the central region of a squared open arena formed by a high black acrylic plastic box (72 cm (L) × 72 cm (W) × 40 cm (H)) for evaluating their explorative behavior in open field for 10 min. During video reviewing, the computer screen image of the squared open arena was divided into 16 square grids. The central 4 square grids were used to represent the central zone of the open field. The total time spent on central zone as “centrality” and the number of squares crossed as “locomotor activities” during the 10 min test was recorded for each animal. A higher level of anxiety-like behavior is marked by decreased centrality and locomotor activities (Gould et al., 2009).

#### Social Interaction Test (SIT)

In each test, two animals that met for the first time were randomly selected from separate cages for evaluating their frequency of social contact or investigation with an unfamiliar conspecific in 10 min. Two animals were firstly introduced to the center of a squared open arena formed by a high black acrylic plastic box (72 cm (L) × 72 cm (W) × 40 cm (H)). During video reviewing, the total number of positive social interaction with partner was recorded for each animal. This included social grooming, smelling, following and crossing the partner. A higher level of anxiety-like behavior is characterized by a lower frequency of positive social interaction (File and Hyde, 1978). The frequency of positive social interaction was additionally used as a behavioral marker of prosocial or affiliative behavior in this study.

#### Forced Swimming Test (FST)

Four hours after the last injection at day 14, a pretest was conducted by placing the animals individually in a cylinder filled with 23 ± 2°C tap water at 30 cm depth for 15 min to induce learned helplessness. A 10 min actual test was conducted 24 h after the pre-test. During video reviewing, the total immobility time and the latency to immobility throughout the 10-min test were recorded. A higher level of depression-like behavior is characterized by increased total time spent immobile and decreased latency to become immobile (Porsolt et al., 1978).

### Tissues Processing and Immunochemistry

Animals were anesthetized with intra-peritoneal injection of overdose sodium pentobarbital (100 mg/kg) and transcardially perfused with normal saline, followed by 4% paraformaldehyde (PFA). After perfusion, adrenal gland of each animal was extracted and weighted. The ratio of adrenal-gland-to-body-weight was calculated to reflect the detrimental effect of CORT treatment on adrenal function. The brains were dissected and post-fixed in 4% PFA overnight and were left in cryoprotection with 30% sucrose. Cryosections with 40 μm thickness were prepared in 1-in-12 series by a cryostat in randomization. Brain sections were affixed on gelatin-coated slides for immunohistochemistry. Antigen retrieval was first carried out in 0.1 M sodium citrate buffer (pH 6.0 at 90°C) for 20 min. BrdU staining was done by incubating the sections in 2 N hydrochloric acid at 37°C for 25 min, followed by neutralization with 0.1 M sodium borax buffer (pH 8.5) at room temperature for 10 min. The primary antibodies were: (1) rat anti-BrdU (1:300; Abcam, Cambridge, MA, USA); and (2) rabbit anti-doublecortin (DCX; 1:200, Cell Signaling Technology, Beverly, MA, USA). Biotinylated goat anti-rabbit secondary antibody (Vector Laboratories, Inc., Burlingame, CA, USA) was applied on sections for peroxidase staining of BrdU and DCX, after overnight incubation with primary antibodies at room temperature. Signal was amplified by avidin–biotin couple (Vector) and visualized with diaminobenzidine (Sigma-aldrich, St Louis, MO, USA). Immunofluorescence staining was done by incubating the sections with both anti-BrdU and anti-DCX antibodies at room temperature overnight, followed by incubation in secondary antibodies (Alexa Fluor 488 goat anti-rat and Alexa Fluor 568 goat anti-rabbit, 1:200, Molecular Probes, Eugene, OR, USA) at room temperature for 2 h.

#### Cell Proliferation Assay

Quantification of BrdU-immunoreactive cells (BrdU-ir) and DCX-immunoreactive (DCx-ir) cells was performed as described previously (Leuner et al., 2012). The BrdU-ir and DCX-ir cells are markers of proliferating cells and immature neurons, respectively. Slides were analyzed by an experimenter blinded to the groupings. The number of immunoreactive cells in the DG was counted on every 12th section of the right brain at 400× magnification. By using unbiased stereology with a semi-automated *StereoInvestigator* (MicroBrightField, Williston, VT, USA) system, quantification of BrdU-ir cells in the hippocampus (sections from 2200 to 4800 μm posterior to bregma) was performed. The parameters for the analysis are as follows: counting frame size as 60 μm × 60 μm, guard zone height as 7.5 μm and dissector height as 15 μm. Six coronal sections were counted per animal. The resulting cell counts were multiplied by 12 in order to obtain unilateral estimation for the total proliferative cell number in the DG (Leuner et al., 2012).

#### Analysis of Dendritic Complexity of Immature Neurons

Sholl analysis was applied to investigate the dendritic complexity (Wang et al., 2008). Ten DCX-ir cells with tertiary dendrites in DG were selected randomly in each animal and they were observed under 400× magnification. Microphotographs were taken and imported into *ImageJ* software (National Institutes of Health, Bethesda, MD, USA) with Sholl analysis plug-in (The Ghosh Lab, UCSD, La Jolla, CA, USA). Concentric circles were drawn on the neurons in every 10 μm radii increment and up to 200 μm away from soma when the neurons were traced in *ImageJ*. Dendritic complexity is assessed based on the numbers of intersections formed by the dendrites and concentric circles. A higher number of intersections indicate a more complex dendritic structure.

### Statistical Analysis

All statistical analyses were conducted with IBM SPSS Statistics version 23 (IBM Corporation, CA, USA). The dataset was firstly analyzed by non-parametric Kruskal-Wallis followed by Dunn-Bonferroni *post hoc* test for pairwise comparison. The physiological, behavioral and cell quantifications were presented in box-plot graphs which the median is used for the central tendency and with the interquartile range of upper 75% and lower 25% percentiles. Repeated measures ANOVA was used to evaluate the changes in dendritic complexity from 10 to 200 μm away from soma of immature neurons in each group. Based on the prior knowledge that the branches of dendrites grow more extensively starting from 50 ± 10 μm away from soma (Po et al., 2015), a two-stage analysis (Tabachnick and Fidell, 2001) was employed to locate the area where between-group difference in dendritic complexity occurred. The level of significance was set at *p* < 0.05. The sampling distribution of each variable was expressed as mean ± standard error of mean (SEM).

## Results

### Effects of Social Isolation and CORT Treatment on Adrenal Gland and Body Weight

**Figure 2:**
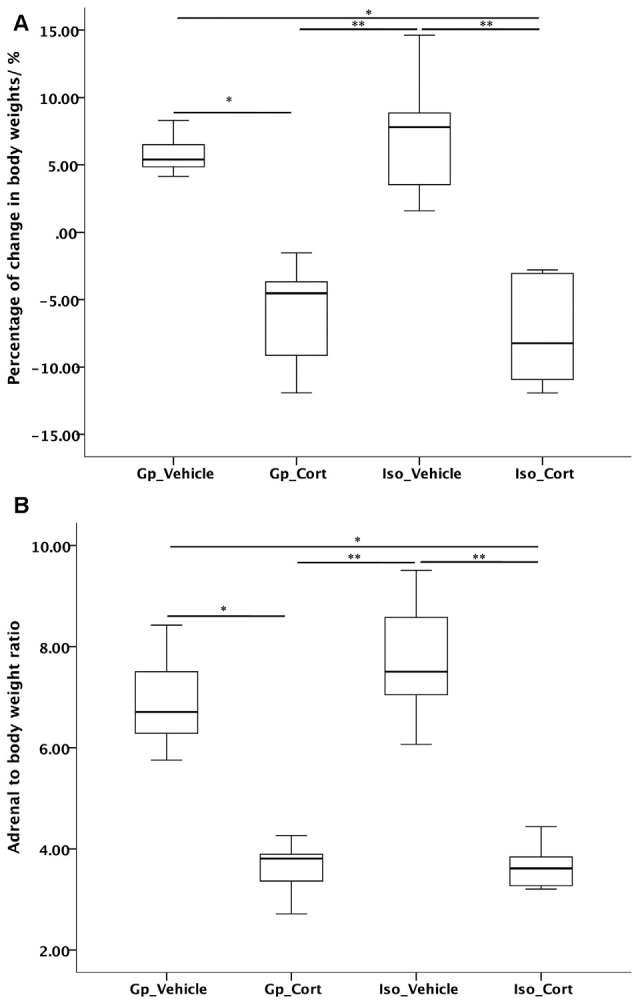
**Effects of social isolation and CORT treatment on (A)** body weight change and **(B)** adrenal-gland-to-body weight ratio in 14 days analyzed by Kruskal-Wallis test followed by Dunn-Bonferroni correction *post hoc* test for pairwise comparison. *p < 0.05, **p < 0.01.

CORT treatment significantly reduced body weight (Figure 2A) and adrenal-gland-to-body-weight ratio (Figure 2B) of rats under both group housing and social isolated condition. Social isolation did not cause significant changes in body weight and adrenal weight ratio under vehicle-treated condition compared to vehicle-treated with group housing condition.

### Effects of Social Isolation and Corticosterone Treatment on Animal Behaviors

#### Depression-Like Behaviors and Anxiety

**Figure 3:**
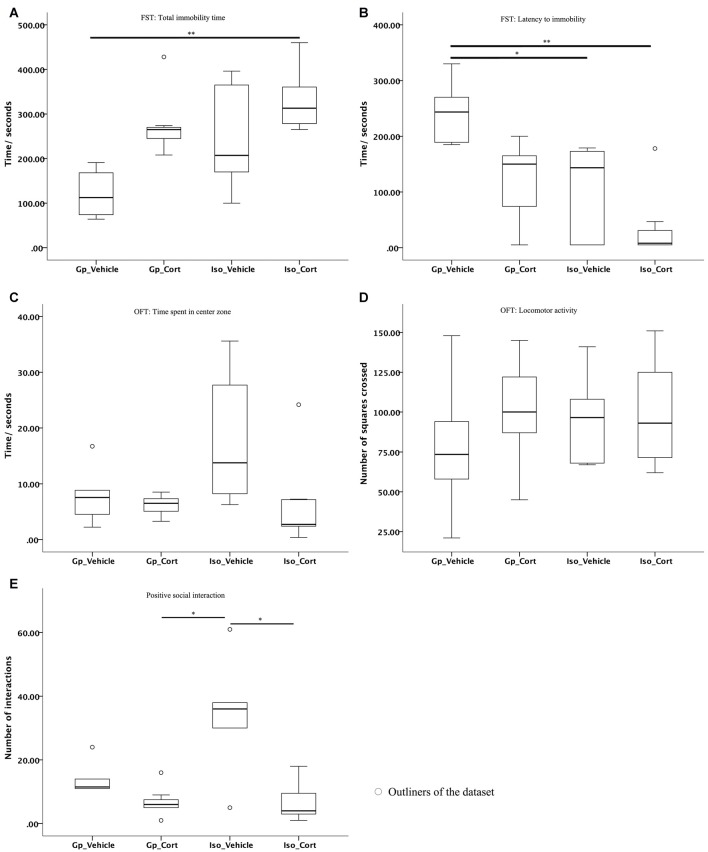
**The latency to immobility and positive social interaction of animals are affected under social isolation with CORT-treated condition. (A)** Mild stress caused by social isolation compounded with CORT-treatment increased total immobility time and **(B)** decreased in latency to immobility in the FST. Furthermore, social isolation only also reduced the latency to immobility time; no significant difference were found in between groups for **(C)** time spent in center zone and **(D)** locomotor activity of the OFT; **(E)** animals with CORT-treated were exhibited decreased positive social interactions in SIT under social isolation. Data were analyzed by Kruskal Wallkis followed by pairwise comparison, *p < 0.05, **p < 0.01; (Gp_Vehicle: *n* = 6; Gp_Cort: *n* = 7; Iso_Vehicle: *n* = 6; Iso_Cort: *n* = 7).

Non-parametric pairwise comparison has shown that only under both social isolated and with CORT-treated conditions (Iso_Cort), the rats were found with increased immobility time (Figure 3A, *p* < 0.01) and decreased latency to immobility compared to the vehicle-treated with group housing animals (Gp_Vehicle; Figure 3B, *p* < 0.01). Furthermore, social isolation (Iso_Vehicle) can decease the latency to immobility compared to the vehicle-treated with group housing animals (Gp_Vehicle; Figure 3B, *p* < 0.01).

The non-parametric pairwise comparison has shown no significant difference between groups for the animal’s performance in the open field test (OFT) in terms of the animals’ time spent in the center of the arena and their locomotor activity (Figures 3C,D).

#### Positive Social Interaction Behaviors

The demonstration of positive social interactions of animals was reduced under both social isolated and CORT-treated condition (Iso_Cort) compared to the social isolated and vehicle-treated animals (Iso_Vehicle; Figure 3E, *p* < 0.01). The Iso_Vehicle animal group demonstrated significantly more positive social interactions than the Gp_Cort animal group (Figure 3E, *p* < 0.05).

### Effects of Social Isolation and CORT Treatment on Hippocampal Neurogenesis

**Figure 4:**
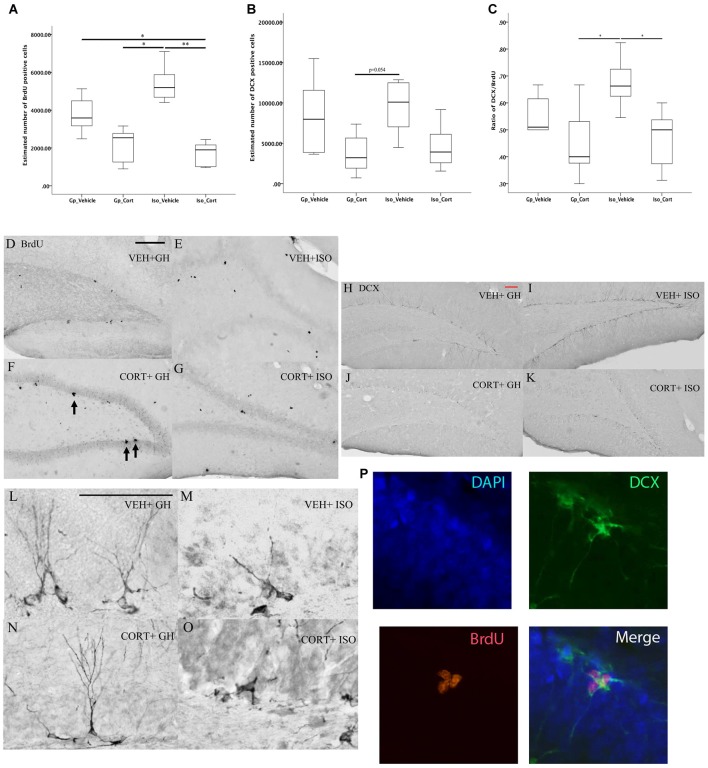
**Social isolation can decrease neurogenesis assay under CORT-treated condition**. Under social isolation, CORT-treatment decreased the number of **(A)** BrdU positive cells and **(C)** the ratio of DCX/BrdU in the hippocampus. This effect could not be observed in the quantification of DCX positive cells only in **(B)**. Data were analyzed by Kruskal Wallkis followed by pairwise comparison, *p < 0.05, **p < 0.01; (Gp_Vehicle: *n* = 6; Gp_Cort: *n* = 7; Iso_Vehicle: *n* = 6; Iso_Cort: *n* = 7). Representative photomicrographs of BrdU-positive cells in Veh + GH **(D)**, Cort + GH **(E)**, Veh + ISO **(F)** and CORT + ISO **(G)** group animal; representative photomicrographs of the quantifications of DCX- positive cells in Veh + GH **(H)**, Cort + GH **(I)**, Veh + ISO **(J)**, CORT + ISO **(K)**; the photomicrographs of the DCX positive cells with 200× magnification of different groups are illustrated in **(L–O)** BrdU positive cell (red) expressing DCX positive cell (green) with DAPI (blue) which is showed by Immunofluorescent photomicrographs **(P)** at 400× magnification. Veh, vehicle-treated; CORT, corticosterone-treated; GH, group housing; ISO, social isolated. Scale bar: 50 μm.

Kruskal-Wallis followed by *post hoc* test for pairwise comparison has shown a significant decreased in the hippocampal proliferating BrdU cells (Figure 4A, *p* < 0.01) and DCX/BrdU ratio (Figure 4C, *p* < 0.05) in CORT-treated rats under social isolated condition (Iso_Cort) compared to the vehicle-treated rats with the same housing condition (Iso_Vehicle). Furthermore, the Iso_Cort animals have significant lesser BrdU positive cells compared the animals with group housing (Gp_Vehicle; Figure 4A, *p* < 0.05). Animals under social isolated condition (Iso_Vehicle) have significant more BrdU positive cells (Figure 4A, *p* < 0.05). And a higher DCX/BrdU ratio (Figure 4C, *p* < 0.05) compared to the group housing CORT-treated animals (Gp_Cort). A marginal difference was found between Iso_Vehicle and the Gp_Cort groups for the quantification of DCX positive cells (Figure 4B, *p* = 0.054) which the Iso_Vehicle group has more DCX positive cells found in the hippocampus. The quantification of the above cell types were summarized in Table 1 and the representative photomicrographs are illustrated in Figures 4D–P.

**Table 1:** **Quantification of BrdU and DCX positive cells**.

Treatments	BrdU positive cells		DCX positive cells		DCX/BrdU ratio	
	Mean	Standard deviation	Mean	Standard deviation	Mean	Standard deviation
Gp_Vehicle	3738.267	959.921	8420.5	4779.842	0.55	0.072
Gp_Cort	2094	929.619	3784.269	2491.186	0.454	0.129
Iso_Vehicle	5403.981	1003.795	9509	3236.947	0.674	0.097
Iso_Cort	1664.771	652.125	4582.857	2756.164	0.467	0.05

### Effect of Social Isolation and CORT Treatment on Dendritic Complexity

**Figure 5:**
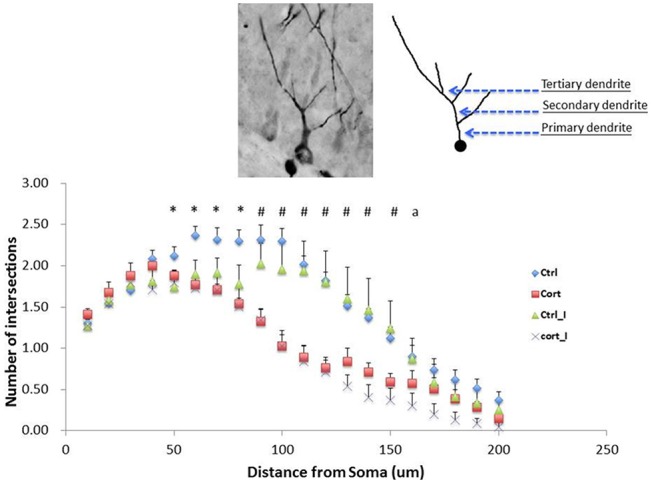
**Main effect of CORT treatment on the dendritic complexity**. Suppression of dendritic complexity was detected from 60 to 200 μm away from soma (*F*_(1,23)_ = 19.99, *p* < 0.001). Values are expressed as mean ± standard error of mean (SEM) and analyzed by one-way ANOVA. *p < 0.05: comparison between Gp_Vehicle with three other groups. ^#^*p* < 0.05: Iso_Vehicle compared with Gp_Vehicle and Iso_CORT respectively. *a* < 0.05: Iso_Vehicle compared with Iso_CORT. Gp, group housing; Iso, social Isolated; CORT, corticosterone-treated.

Repeated measure ANOVA was conducted using the number of intersections formed by dendrites of immature neurons on concentric circles as dependent variable. The within-subjects variable was the distance measured from 10 μm to 200 μm, a total of 20 data points, away from soma, while the between-subjects factors were housing condition and treatment. The results indicated that a higher complexity of dendritic branches was found in vehicle-treated with group housing from 50 μm to 80 μm away from soma compared to other treatment groups. From 90 μm to 150 μm away from soma, significant decrease in dendritic complexity was found in both CORT-treated groups under both living conditions compared to the vehicle-treated with isolation group. Further at 160 μm away from soma, CORT-treated with social isolation has shown a significant decrease in dendritic complexity compared with the vehicle-treated with social isolation group (Figure 5).

## Discussion

The present study examined the effects of social isolation on hippocampal neurogenesis and emotional behaviors in a model of hypercortisolemia. This study has provided evidence that CORT treatment can decrease the positive social interaction and affects hippocampal neurogenesis in social isolated rats. Though we have shown that social isolation alone can affect the latency to immobility in FST, it did not suppress hippocampal neurogenesis as expected. The discrepancies found among different studies are discussed in the following.

### Social Isolation Induced Depression-Like Behavior but Not Anxiety-Like Behavior and the Enhancement of Neurogenesis

This study showed that social isolation has the effect of decreasing latency in immobility in FST. However, our findings did not support the hypothesis that social isolation increased anxiety-like behavior in OFT and social interaction test (SIT). Such phenomenon could be associated with the potentially more adaptive ability of the adult rats recruited in this study in response to an unfamiliar environment in an open arena and meeting with conspecifics in such a social environment. Therefore, they responded differently from those younger rats which displayed anxiety-like-behavior in OFT (Djordjevic et al., 2008; Lukkes et al., 2009) and SIT (Lukkes et al., 2009; Shoji and Mizoguchi, 2011). In addition, this study utilized a 14-day social isolation paradigm. It differed from a study which reported that elevated HPA axis activity is implicated in high level of distress among male Wistar rats after a prolonged social isolation period of 21 days (Lukkes et al., 2009). In other words, social isolation for 14 days may represent a comparatively mild stress, especially for adult rats. Therefore, they might be able to withstand the stressors in the unfamiliar environments of both OFT and SIT. Although our current study showed that social isolation under CORT-treated condition can induce immobility in FST which has been commonly regarded as an indicator of depression-like behavior. It should be noted that the immobility behaviors demonstrated in rodents could be dependent on different baseline levels among different strains and genetic background (Powell et al., 2012). In addition, the immobility and social interaction performance in these tests may be due to panic or agitation as demonstrated among individuals. There is a limitation in this study that only FST was applied for measuring the depressive-phenotypes. Some other behavioral tests, such as tail suspension test, will be needed alternatively to further validate the depressive-phenotypes demonstrated in rodents.

### Contradictory Effects of Social Isolation on Emotional Behavior and Neurogenesis

The effects of social isolation has been previously demonstrated to induce depression-like behaviors in rodents which has been reported by other groups to be associated with altered hippocampal neurogenesis (Famitafreshi et al., 2015). Though the current study demonstrates that social isolation can reduce the latency to immobility in FST, current results did not demonstrate the reduction in neurogenesis by social isolation alone. A possible explanation is that neurogenesis may not solely contribute to the etiology of depression. A few studies have reported that the ablation of hippocampal neurogenesis in rodents did not induce any depression-like or anxiety-like behavior in rodents (Santarelli et al., 2003; Sahay and Hen, 2007). This indicates that the alterations of neurogenesis may not be directly correlated to the development of emotional or anxiety-like behavior. Indeed, the heterogeneity of findings on depression have implied that the dysfunction of several brain regions or other factors may be involved and disease development may be independent of hippocampal neurogenesis. For example, a recent report has shown that the impairments in behavioral changes induced by social isolation are associated with the alteration of myelination and related glial cells in the prefrontal cortex (Liu et al., 2016). This has provided a novel direction of changes in glial cells associating with depression.

Another possible explanation is due to the animals’ housing condition that we have adopted in the current experiments. Social isolated animals were housed individually in cages with dimension of 43 × 27 × 16 cm whereas group-housed animals were kept in groups of 3–4 animals in cages with same dimension. The group cages in this study may possibly be overcrowded and thus increase stress among animals where less space is available for each individual. It is thus suspected that the above housing issue may possibly counterbalance the possible beneficial effects of a group housing condition over the social isolated condition.

### Social Isolation Stimulated Positive Social Interactions with Conspecifics

The present study showed that mild stress induced by social isolation for 14 days resulted in an increased frequency of positive social interaction with conspecifics among the vehicle-treated animals. In fact, the result parallels the findings of an increased frequency of affiliative behaviors with conspecifics after social isolation for 6 weeks among adult female prairie voles (Lieberwirth et al., 2012). It possibly reflects that SD rats and prairie voles, which are both social species, might have an intrinsic need of finding companions through social exploration or investigation in order to reduce anxiety (Taylor, 2006; Lieberwirth and Wang, 2012). In line with this suggestion, several studies have also indicated a stimulation of prosocial behavior in terms of increased frequency of social interaction or affiliation after a prolonged period of social isolation in juvenile male Wistar rats (Tanaś et al., 2015) and young adult male F344/N rats (Shoji and Mizoguchi, 2011).

## Conclusion

In summary, this study has provided preliminary evidence that normal adult rats responded to a socially isolated environment with increase in dendritic plasticity that indicated a higher level of neuroplasticity. Under this mild stress condition, the enhanced neuroplasticity may underlie the adaptive behaviors such as social affiliation or bonding with conspecifics. However, this innate protective mechanism, or resilience toward adversity, may be compromised in depression.

## Author Contributions

All authors: design of experiment and data collection; analysis of result; manuscript proof reading and approval for submission.

## Conflict of Interest Statement

The authors declare that the research was conducted in the absence of any commercial or financial relationships that could be construed as a potential conflict of interest.
